# Effect of mucosal incision on postoperative olfactory function in the endoscopic transsphenoidal approach to pituitary adenoma

**DOI:** 10.3389/fneur.2026.1853732

**Published:** 2026-07-22

**Authors:** Ruixiang Wei, Lang Long, Moukun Liu, Jinghuan Liu, Yuanliang Ye, Jiamin Qin

**Affiliations:** 1 Department of Neurosurgery, LiuZhou People's Hospital, Affiliated to GuangXi Medical University, Liuzhou, Guangxi, China; 2 Liuzhou Neuroendoscopic Anatomy and Clinical Application Technology Engineering Research Center, Liuzhou, Guangxi, China; 3 The First People’s Hospital of Yulin City, Guangxi, China

**Keywords:** neuroendoscopy, neurosurgery, olfactory function, pituitary adenoma, transnasal transsphenoidal approach

## Abstract

**Objective:**

This study aims to evaluate the impact of different mucosal incision techniques during endoscopic transnasal transsphenoidal surgery on postoperative olfactory function in patients.

**Methods:**

A retrospective analysis was conducted on clinical data of patients with pituitary adenomas, treated with endoscopic transnasal transsphenoidal surgery at the Neurosurgery Department of Liuzhou People’s Hospital from December 2024 to October 2025. Patients were divided into the mucosal flap group and the submucosal group based on the nasal septum mucosal incision technique used. Postoperative olfactory dysfunction was assessed using a five-flavor odor test and an olfactory function subjective evaluation questionnaire.

**Results:**

The incidence of olfactory dysfunction was 60% (46/76), 36% (27/76), and 17% (13/76) at one, three, and six months after surgery. Postoperatively, the submucosal group exhibited better recovery of olfactory function than the mucosal flap group at one month (*p* = 0.02) and three months (*p* = 0.03). However, no significant difference was observed between the two groups six months after surgery (*p* = 0.66). The submucosal group showed significantly greater sensitivity to foul odors at 1 and 3 months postoperatively relative to the mucosal flap group (*p* = 0.02 and *p* = 0.03, respectively).

**Conclusion:**

Endoscopic transnasal transsphenoidal surgery for pituitary adenomas results in short-term olfactory dysfunction. Submucosal incisions in the nasal septum mucosa facilitate faster and earlier recovery of odor recognition. In cases involving large pituitary adenomas or complex surgical procedures, the mucosal flap approach is preferred; there is no significant difference in long-term olfactory function following surgery.

## 1 Introduction

The human central nervous system is frequently affected by a pituitary adenoma, a primary benign tumor. Among intracranial tumors, pituitary adenomas rank third in incidence after glioma and meningioma, accounting for approximately 10–15% of all cases. It has a substantial clinical effect and significantly shortens the operative time by employing neuroendoscopy to diagnose these tumors through the nasal cavity and sphenoid sinus (1). Additionally, this procedure significantly improves the visual field and alleviates hormonal dysfunction following tumor removal (2, 3). During the surgery, the sphenoid sinus can be approached using three distinct techniques: The mucosal flap approach to the nasal septum, the submucosal approach to the nasal septum, and the double nostril approach. The primary difference among these methods lies in the manner of nasal mucosal incision. In the submucosal approach, the nasal septum is accessed by incising the septal mucosa or the anterior wall of the sphenoid sinus. In contrast, the mucosal flap approach involves creating a nasal septum mucosal flap, while the double nostril approach requires the excision of the mucosa on both sides of the posterior nasal septum. The submucosal approach has numerous benefits, including reduced trauma, quicker recovery, and fewer complications (4). However, it is important to consider the following: the limited surgical visual field, the need for a specialized surgeon, and the extended time required to complete the operation. The nasal septum mucosal flap approach offers a more extensive range of applications, superior vision, shorter operation time, and more spacious free operating space (5, 6).

Following neuroendoscopic surgery through the nasal cavity and sphenoid sinus, patients may experience varying levels of olfactory sensation dysfunction, which may adversely affect their daily lives (7, 8). For instance, the loss of smell and taste can decline the quality of life by impairing the ability to determine food freshness. Additionally, the risk related to food safety and environmental hazards may increase (9), as the patient may be unable to promptly identify gas leaks, fires, or other threats, and lack early warning capabilities for potentially hazardous gases (10). Restricted social and professional activities, financial strain of possible medical costs, and extra safety gear might result in psychological issues (11). In this study, individuals with pituitary adenoma were convened from Liuzhou People’s Hospital’s Department of Neurosurgery. Their postoperative olfactory impairment and recovery after retrospective analysis based on various mucosal incision techniques were examined. This study aimed to explore theoretical support for surgical techniques used to treat pituitary adenomas.

## 2 Materials and methods

### 2.1 The topic under investigation

Medical records of 76 patients who had endoscopic transsphenoidal surgery for pituitary adenomas at Liuzhou People’s Hospital’s Department of Neurosurgery between December 2024 and October 2025 were collected for the study. These patients were divided into an mucosal flap group and a submucosal group based on various incision techniques employed during the procedure. This study was approved by the Ethics Committee of Liuzhou People’s Hospital (Approval Number: 2024SL023-ZGC).

This retrospective observational study was approved by the Institutional Ethics Committee of Liuzhou People’s Hospital on April 2024 (approval number: 2024SL023-ZGC). Serial olfactory function examinations including the five-flavor odor identification test and the Smell Disorder Questionnaire (SODQ) administered at 1, 3, and 6 months postoperatively were custom-designed exclusively for this research and do not constitute part of the hospital’s standard postoperative follow-up protocol for patients with pituitary adenoma. No research-related olfactory assessments or structured questionnaire surveys were performed before ethical clearance was granted. Following approval, investigators retrospectively screened all patients who underwent endoscopic endonasal transsphenoidal resection between December 2024 and October 2025 and contacted eligible subjects to complete unified standardized olfactory testing. All patient identifiers were permanently de-identified prior to data aggregation and statistical analysis. All eligible participants signed written informed consent prior to any study-related olfactory assessments.

### 2.2 Inclusion criteria

(1) Patients aged between 18 and 65 years; (2) patients with confirmed pituitary adenoma through postoperative pathological examination involving comprehensive preoperative imaging, endocrinology, and postoperative follow-up data; (3) patients having normal nasal cavity and sphenoid sinus structure before surgery; (4) patients whose tumor was removed using neuroendoscopy through the nasal cavity and sphenoid sinus; (5) patients having good olfactory function before surgery; (6) patients who called for or sought outpatient care following surgery; (7) patients who signed an informed consent form.

### 2.3 Exclusion criteria

(1) Patients who have had transsphenoidal or craniotomy for suprasellar tumor removal by microscope; (2) patients who have been diagnosed with craniopharyngioma, germ cell tumor, or langerhans cell histiocytosis; (3) patients having severe sinus and nasal infection; (4) patients with a congenital anomaly in the paranasal sinus structure(severe congenital nasal septal deviation, congenital nasal septal perforation, aplastic or non-aerated sphenoid sinus, congenital atresia of ethmoid or maxillary sinus, congenital narrow olfactory cleft, and bony defects of the olfactory cribriform plate. All patients received preoperative thin-section sinus CT and pituitary MRI, and two independent radiologists reviewed all imaging data to screen for the above malformations); (5) patients with radiotherapy history; (6) patients with severe preoperative anosmia; (7) patients with incomplete follow-up information.

### 2.4 Grouping basis

**Figure 1:**
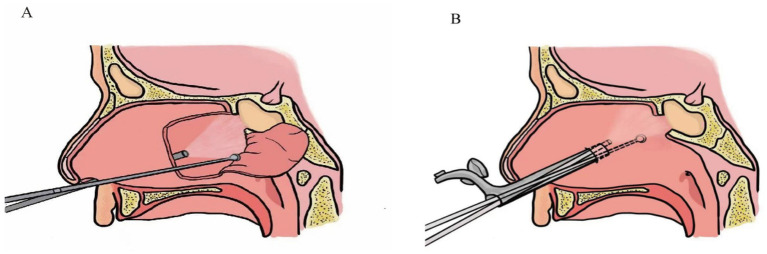
Presenting different methods about nasal septum mucosa. **(A)** Mucosal flap group; **(B)** Submucosal group.

The patients were divided into mucosal flap group and submucosal group based on the mucosal incision technique used. For the mucosal flap group, an arc-shaped incision was made in the nasovestibular mucocutaneous junction, 2–3 mm inferior to the sphenoid sinus ostium and along the posterior perpendicular plate of the nasal septum (Figure 1A). For the submucosal group, a vertical incision was created in the unilateral nasal cavity by incising the nasal septal mucosa, with subsequent horizontal extension along the anterior surface of the middle turbinate for approximately 2 cm (Figure 1B). No preoperative stratification was performed according to tumor size or surgical complexity, and the selection of mucosal incision technique only depended on the operating habits of individual neurosurgeons. Baseline data including tumor diameter, operation time and intraoperative blood loss were balanced between the two groups (all *p* > 0.05), which suggested low risk of selection bias in this retrospective cohort. The mucosal flap approach requires a large arc-shaped incision with extensive full-thickness dissection of nasal septal mucosa, which substantially damages the blood supply of olfactory epithelium around the olfactory cleft and creates a wide mucosal defect. In contrast, the submucosal approach only involves a shallow vertical mucosal cut with limited submucosal separation without complete mucosal flap elevation, which preserves the integrity of olfactory cleft mucosa, olfactory fila and olfactory epithelium. Such anatomical differences lead to faster recovery of odor recognition, within the first 3 months after surgery, while mucosal self-repair eliminates intergroup differences in olfactory function at 6 months postoperatively.

### 2.5 Operative procedure

**Figure 2:**
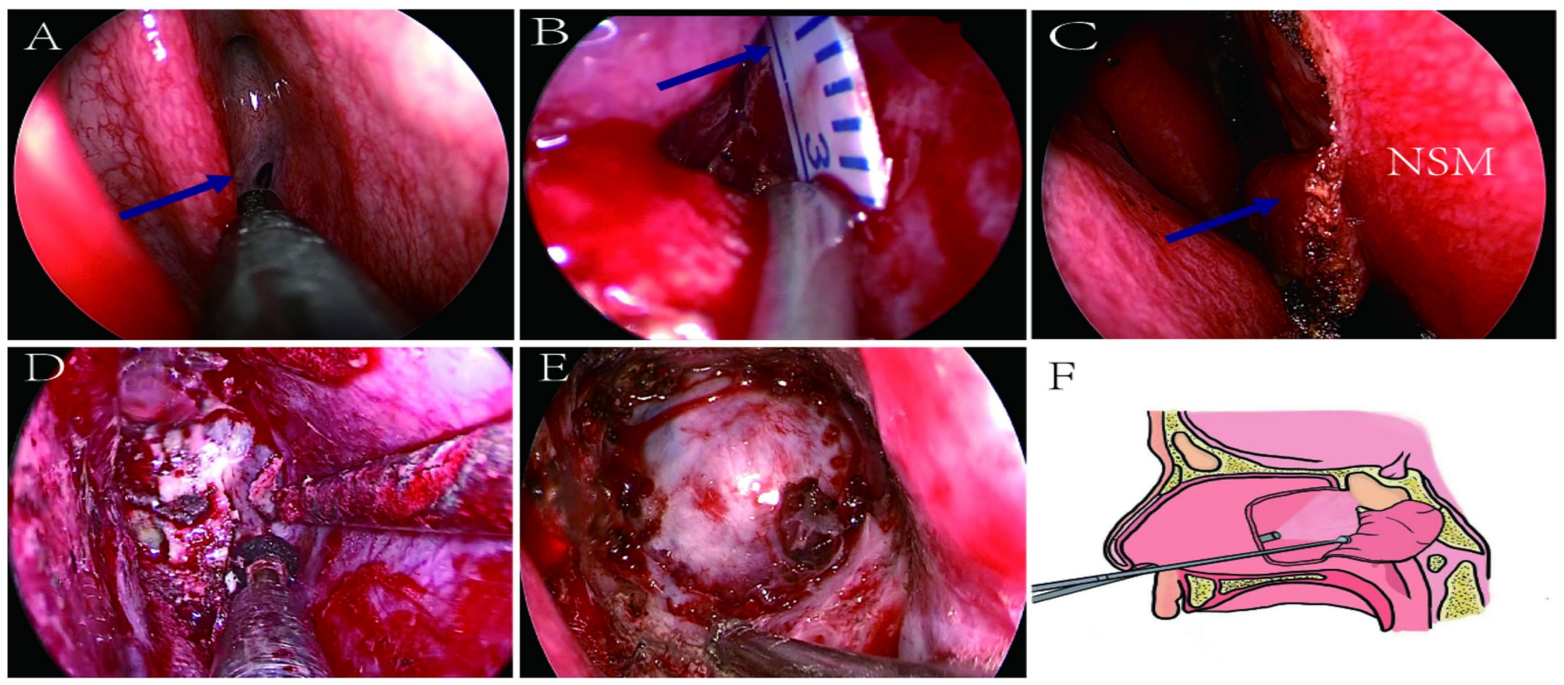
Neurosurgical endoscopy via the nasal and sphenoid sinus is used to treat pituitary adenomas with a mucosal flap approach. **(A)** The opening site of the sphenoid sinus; **(B)** nasal septum mucosa incision at the 1/3 point; **(C)** nasal mucosal flap production, peeling of nasal septum mucosa; **(D)** exposure of the sella floor; **(E)** exposure of the dura mater at the base of the skull; **(F)** incision 2 cm away from the sieve top.

**Figure 3:**
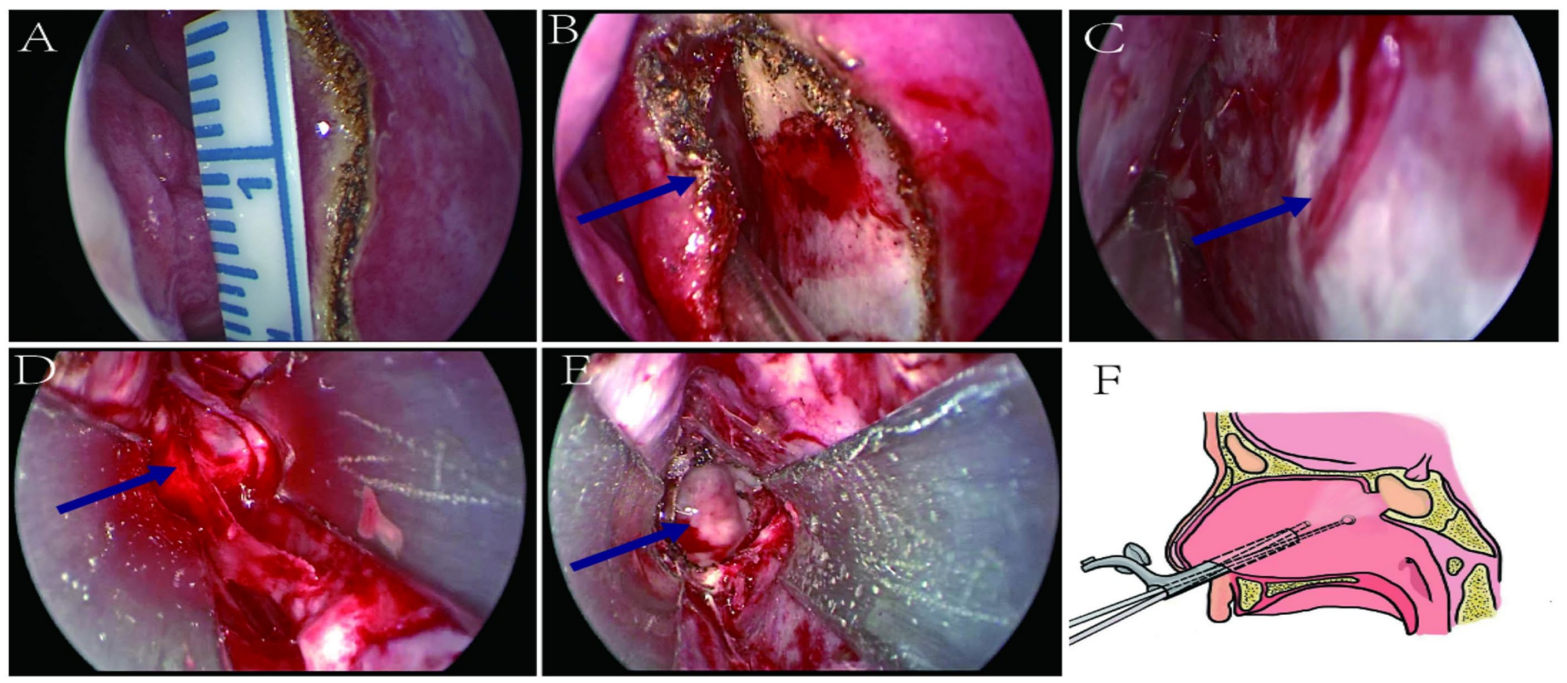
Neurosurgical endoscopy via the nasal and sphenoid sinus is used to treat a submucosal approach to a pituitary adenoma. **(A)** Mucosal incision; **(B)** site of mucosal sampling from the nasal septum; **(C)** the removal of nasal septum mucosa; **(D)** exposure of the anterior wall of the sphenoid sinus and nasal septal cartilage; **(E)** insertion of the expander and exposure of the anterior wall of the sphenoid sinus; **(F)** exposure of the base of the epidural.

The sphenoid sinuses and nasal cavity were sufficiently exposed by tilting the head backward at an angle of 10° ~ 15°. Following general anesthesia, the patient’s computed tomography and magnetic resonance imaging data were imported into the neuronavigation system to register the case information for the three-dimensional reconstruction of the tumor, intracranial artery, and sphenoid region anatomy. Before the surgery started, cotton-sliver, which contains 0.01% epinephrine and physiological saline, was placed into the bilateral nasal cavities to compress the nasal mucosa. The nasal septum mucosal flap (Figure 2) was made, or a simple nasal septum incision (Figure 3) was performed by a high-frequency electrosurgical knife under endoscopy. To expose the anterior wall of the sphenoid sinus, the bony nasal septum was removed to connect the left and right nasal cavities. Using a micropower system, the following procedures were conducted under neuroendoscopy: Removing the remaining anterior wall of the sphenoid sinus, enlarging the bone window to 2.0 × 2.0 cm, exposing the sella turcica floor, and biting off the bone of the sella turcica floor to reveal the dura mater. During image-guided navigation, the bilateral internal carotid arteries were first identified and carefully avoided to prevent iatrogenic injury. The dura mater was then electrocauterized for hemostasis, punctured, and incised in a “cross” pattern; the incision was further expanded using electrocautery to adequately expose the surgical field. Concurrently, the spectrum of pituitary space-occupying lesions was characterized, and the tumor exhibited a grayish-red gross appearance. The bottom, lateral, and upper portions of the tumor were gradually dissected and removed under endoscopy. Tumor samples were sent for pathological analysis after tumor excision around the optic nerve to reduce pressure. Procedures were performed until the diaphragma sellae was visible, and malignancies were removed. The base of the skull was repaired with a 4 × 3 cm dura mater patch and cemented with gelatin. On the base of the skull, gelatin sponge was spread out flat, and an expanded sponge was used to create double-sided packing.

### 2.6 Olfactory function assessment

The five-flavor olfactory test solution created by the Institute of Semiconductors, Chinese Academy of Sciences, was used in this study as a subjective test to assess the postoperative olfactory function of patients at one, three, and six months following surgery. Sour, Fruity, floral, chilly, and unpleasant smells were symbolized using acetic acid, pentyl acetate, eugenol, menthol, and 3-methylindole, respectively. Five concentrations of varying orders of magnitude were created by diluting each olfactory component 10 times its weight concentration. Each olfactory solution was categorized into 1, 2, 3, 4, and 5 concentrations, with 1 denoting the lowest. As blank controls, three bottles of liquid paraffin were prepared. Acetic acid (a, sour), pentyl acetate (b, fruity), menthol (c, cool), eugenol (d, floral), and 3-methylindole (e, fetor) were used as odor stimuli 3-methylindole. Each participant typically needed 3–5 min to complete the entire olfactory testing procedure. The subjective olfactory function of the patient was indicated by their recognition threshold. The patient’s total olfactory recognition threshold was represented by the average recognition threshold for five different kinds of olfactory compounds. Normal olfactory function was indicated by a recognition threshold score of < 3, whereas aberrant olfactory function was indicated by a score of > 3. The five-flavor odor test solution was developed by the Institute of Semiconductors, Chinese Academy of Sciences, and its reliability and validity have been fully verified in Chinese adult populations, which has been widely used in domestic clinical studies focusing on sinonasal and skull base surgery-related olfactory dysfunction. This test simultaneously evaluates two indicators: odor identification ability and olfactory recognition threshold. The average threshold value of five odors was calculated as the comprehensive olfactory outcome indicator for statistical analysis.

The olfactory function subjective evaluation questionnaire was used to quantitatively assess the olfactory function, with a scale from 0 to 3 (0 = “strongly disagree”; 1 = “somewhat disagree”; 2 = “somewhat agree”; 3 = “strongly agree”). At one, three, and six months after surgery, olfactory function was quantitatively assessed using the olfactory dysfunction questionnaire (SODQ) (6). Normal olfactory function was indicated by a score of 22 or higher, whereas olfactory dysfunction was indicated by a score of 22 or lower. All olfactory function tests and SODQ questionnaire assessments were completed by physician who were blinded to the patients’ surgical grouping information, and single-blind evaluation was adopted to reduce assessment bias. The Smell Disorder Questionnaire (SODQ) used in this study has completed Chinese cultural adaptation, translation and standardized reliability and validity verification for Chinese patients undergoing sinonasal skull base surgery.

### 2.7 Follow-up protocol

Following discharge, telephone follow-up and outpatient review were used to collect information on the patients’ olfactory function. All telephone interviews and outpatient olfactory examinations were designed specifically for this research rather than routine clinical follow-up protocols.

### 2.8 Statistical methods

Statistical data were analyzed using the Statistical Package for the Social Sciences software (version 26.0), and statistical graphics were made using the GraphPrism software (version 8). Categorical data are reported as percentages (%), whereas measurement data are displayed as mean ± standard deviation (x ± s). Fisher’s exact probability or the chi-square test was used for group comparisons. T-tests were used to compare groups for continuous variables. A *p* value < 0.05 was considered statistically significant for all analyses.

## 3 Results

A total of 76 individuals with pituitary adenomas were included in this study.

All patients were categorized into two groups: The submucosal group (30 cases) and the mucosal flap group (46 cases). The average age of the 30 females and 16 males in the mucosal flap group was 49.93 ± 1.64 years, whereas that of the 15 males and 15 female members of the submucosal group was 46.23 ± 2.44 years.

No statistically significant differences were observed between the two groups in terms of gender (*p* = 0.18), age (*p* = 0.19), surgical time (*p* = 0.36), tumor diameter (*p* = 0.25), blood loss (*p* = 0.31), hypertension (*p* = 0.31), diabetes (*p* = 0.49), and renal insufficiency (*p* = 0.67) (Table 1).

**Table 1:** Comparison of general data between two groups of patients with pituitary adenomas.

	Mucosal flap group (*n* = 46)	Submucosal group (*n* = 30)	χ^2^	*p*
Sex			1.74	0.18
Male	16	15		
Female	30	15		
Age (y)	49.93 ± 1.64	46.23 ± 2.44	1.25	0.19
Tumor diameter(mm)	26.15 ± 1.33	29.73 ± 2.16	0.91	0.36
Operation time(min)	170.00 ± 49.00	152.15 ± 68.10	1.14	0.25
Amount of bleeding(mL)	82.17 ± 10.95	73.83 ± 12.58	1.0	0.31
Complication				
Hypertension	22	16	1.01	0.31
Diabetes	8	6	0.47	0.49
Renal inadequacy	6	4	0.17	0.67

### 3.1 Comparison of postoperative olfactory dysfunction in two groups of patients with pituitary adenomas

Approximately 46 (60%) of all patients with pituitary adenoma surgery displayed olfactory impairment within one month of the procedure. Olfactory impairment was observed in 27 patients (36%) at three months after surgery, while 13 patients (17%) continued to experience olfactory impairment at six months. Olfactory impairment was observed in 71.74, 47.82, and 17.39% of cases in the mucosal flap group, within one, three, and six months of surgery, respectively. Within a month, 43.33% of the submucosal group experienced olfactory impairment, followed by three (16.67%) and six (16.67%) months. After one and three months of surgery, the submucosal group manifested a lower incidence of olfactory impairment than the mucosal flap group. However, after six months, the incidence was comparable (Table 2).

**Table 2:** Comparison of the occurrence of olfactory dysfunction in two groups of patients with pituitary adenomas at different times after operation.

Group	Olfactory dysfunction (%)		
	One month after surgery	Postoperative 3 months	Six months after surgery
Mucosal flap group	71.74 (33/46)	47.82 (22/46)	17.39 (8/46)
submucosal group	43.33 (13/30)	16.67 (5/30)	16.67 (5/30)

### 3.2 Olfactory recognition thresholds of the two groups were compared at different time points

There was no statistically significant difference between the two groups in preoperative recognition threshold. After one (*p* = 0.01) and three months (*p* = 0.04) of surgery, the olfactory identification threshold of the submucosal group was significantly lower than that of the mucosal flap group. The olfactory recognition thresholds between the two groups were statistically nonsignificant at six months after surgery (*p* = 0.68) (Table 3).

**Table 3:** Comparison of olfactory recognition threshold between the mucosal flap group and the submucosal group at different postoperative time points.

Dimension	Time	Mucosal flap group	Submucosal group	*t*	*p*
Recognition threshold	Preoperative	2.20 ± 0.40	2.20 ± 0.40	0.00	*p* > 0.99
Recognition threshold	Postoperative 1 month	4.60 ± 1.30	3.80 ± 1.60	2.392	0.01
Recognition threshold	Postoperative 3 months	3.20 ± 1.90	2.87 ± 1.30	2.042	0.04
Recognition threshold	Postoperative 6 months	2.80 ± 1.00	2.70 ± 1.10	0.407	0.68

### 3.3 The two groups were compared in the subjective evaluation questionnaire of olfactory function at different time points

The olfactory function scores of the mucosal flap group were significantly lower than those of the submucosal group at one and three months following the procedure (*p* = 0.00 and *p* = 0.00, respectively). Six months following surgery, no significant difference was observed in the subjective assessment of olfactory function between the two groups (*p* = 0.66) (Table 4).

**Table 4:** Comparison of olfactory scores at different time points after surgery in patients with pituitary adenomas.

Time	Mucosal flap group	Submucosal group	*t*	*p*
Postoperative 1 month	30 ± 15	18.5 ± 20	2.692	0.00
Postoperative 3 months	22 ± 13	12 ± 16	2.861	0.00
Postoperative 6 months	8 ± 11	7 ± 9	0.431	0.66

### 3.4 Degree of five taste olfactory solutions was compared at different time points in two groups

**Figure 4:**
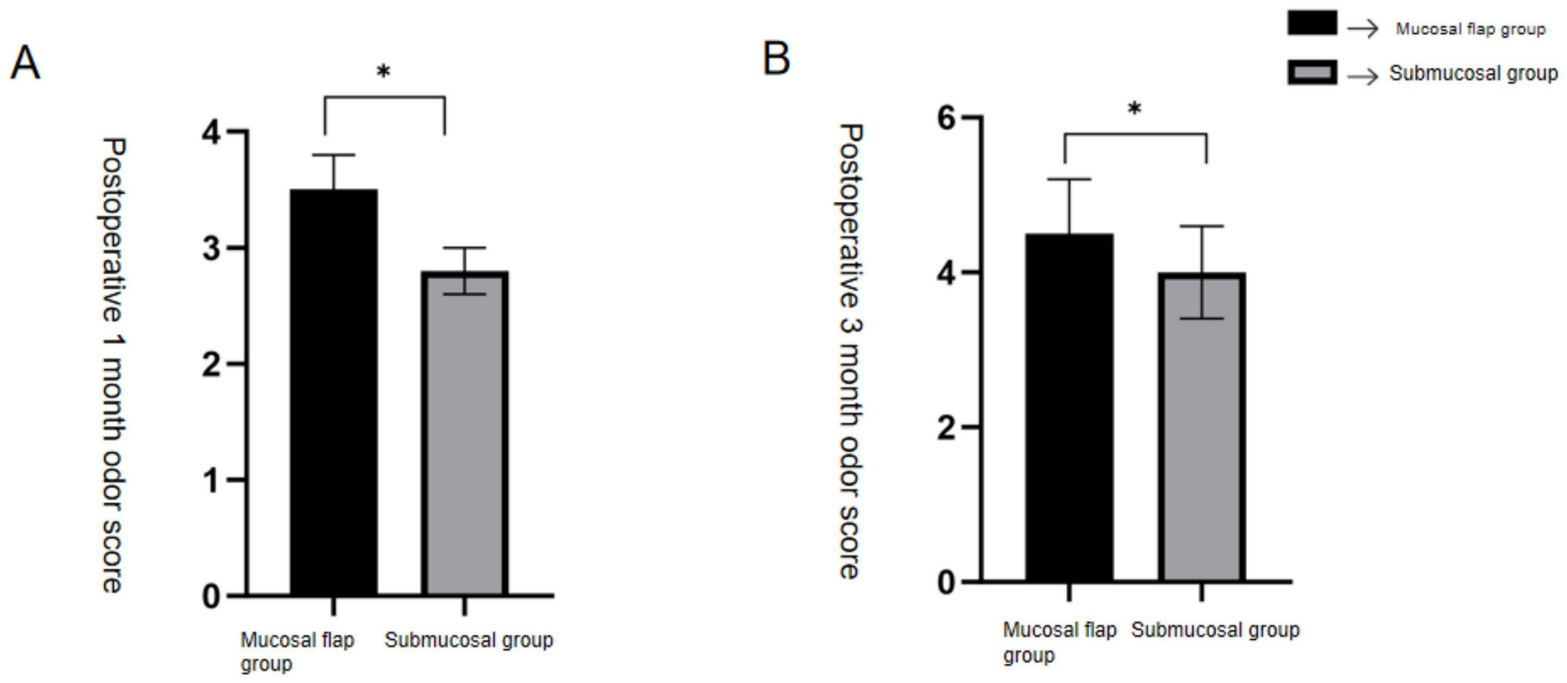
Bar charts of olfactory recognition thresholds at postoperative time points. **(A)** 1 month postoperatively; **(B)** 3 months postoperatively. *indicates statistically significant intergroup difference with *P* < 0.05 at the corresponding postoperative time point.

At various intervals following surgery, the two groups were compared for five odors: acidity, fruity, flower, cool and refreshing, and fetor. There was no statistically significant difference between the two groups when comparing the other four scents (*p* > 0.05). Only fetor recognition showed significant intergroup differences at 1 and 3 months postoperatively (*p* = 0.02, *p* = 0.03). Patients in each group experienced a noticeable improvement in their odor when compared to three months postoperatively, although it had not yet returned to its preoperative state. Three months after surgery, there were no statistically significant differences between the two groups in terms of cool and refreshing (*p* = 0.25), flower (*p* = 0.45), fruity (*p* = 0.40), and acidity (*p* = 0.35). The odor threshold at three months after surgery was lower than that observed at one month after surgery, suggesting that the olfactory function of both groups had improved. Patients’ olfactory function continued to improve six months after surgery, with some odor recognition thresholds reaching their preoperative levels. All identification thresholds had returned to normal, although there were no statistically significant differences between the two groups in the five smells (*p* > 0.05) (Figure 4; Table 5).

**Table 5:** Degree of five taste olfactory solutions was compared between the mucosal flap group and the submucosal group at different time points.

	Mucosal flap group			Submucosal group		
	Postoperative 1 month	Postoperative 3 months	Postoperative 6 months	Postoperative 1 month	Postoperative 3 months	Postoperative 6 months
Acidity	3.5 ± 0.4	3.3 ± 0.4	3.0 ± 0.3	3.2 ± 0.3	3.0 ± 0.3	2.8 ± 0.2
Fruity	4.5 ± 0.6	4.2 ± 0.6	3.8 ± 0.5	4.0 ± 0.5	3.8 ± 0.5	3.5 ± 0.4
Flower	4.8 ± 0.7	4.5 ± 0.7	4.0 ± 0.6	4.2 ± 0.6	4.0 ± 0.6	3.7 ± 0.5
Cool and refreshing	4.2 ± 0.5	3.6 ± 0.4	3.2 ± 0.3	4.2 ± 0.5	4.0 ± 0.5	3.5 ± 0.4
Fetor	3.5 ± 0.3	3.2 ± 0.3	2.8 ± 0.2	2.8 ± 0.2	2.6 ± 0.2	2.5 ± 0.2

## 4 Discussion

The postoperative olfactory function of 76 patients with pituitary adenomas who underwent neuroendoscopic transsphenoidal surgery was evaluated. The results indicated that the overall prevalence of olfactory dysfunction was 60% at one month, 36% at three months, and 17% at six months. Compared to the septal flap route, the submucosal method resulted in a higher subjective evaluation score for early olfactory function in patients. Their early olfactory function also recovered more rapidly than that of patients who underwent the septal mucosal flap approach. The subjective evaluations of olfactory function of patients who underwent the submucosal method and those who underwent the mucosal flap approach did not differ at six months following the surgery. Conversely, the submucosal technique induces an increased sensitivity to odor.

The olfactory function is a critical component of daily existence, providing essential information, warning of potential dangers, and contributing positively to emotional well-being and work performance. Doubtlessly, olfactory dysfunction declines the quality of life, ultimately resulting in the brain processing of negative emotions in a disorder, which is susceptible to melancholy. This, in turn, results in the development of other severe mental illnesses (12). Zada et al. reported a 9% incidence of postoperative olfactory dysfunction. These findings indicated that the endoscopic transsphenoidal approach resulted in less mucosal injury, and the short-term olfactory dysfunction was primarily due to nasal packing.

Several explanations for postoperative olfactory dysfunction have been proposed, including the possibility that the narrowed nasal cavity may obstruct the flow of odorants to the olfactory receptors, airflow may be mechanically diverted from the olfactory region, or local airflow changes may occur near the olfactory fissure. The inhaled air may be diverted away from the olfactory region as the nasal cavity beneath the middle turbinate expands, resulting in olfactory dysfunction (13, 14).

Dusick et al. performed a neuroendoscopic rhinoscopic approach to treat 259 patients, revealing that the incidence of postoperative olfactory dysfunction is approximately 10% (15). The rate of postoperative olfactory disorder complications associated with endoscopic pituitary surgery was approximately 9.5%, as summarized by Charalampaki et al. in a retrospective analysis of 150 patients (16). Furthermore, Netuka et al. conducted a prospective study involving 143 patients and revealed a 9.8% incidence of postoperative olfactory dysfunction. They recommended minimizing the risk of olfactory deterioration when the cranium base is reconstructed using an endoscopic nasal approach without a mucosal flap. However, additional research is necessary to conduct an objective assessment of olfactory function.

This study indicated 60.5% (46/76) and 35.5% (27/76) incidence of olfactory dysfunction at one and three months after surgery, respectively. It is a significant decrease from the previous time point. However, at six months after surgery, the incidence of olfactory dysfunction further decreased to 17.1% (13/76).

The postoperative comprehensive olfactory score showed no significant intergroup difference between the submucosal group and mucosal flap group, as reported in previous studies (15). Additionally, the nasal mucosa injury of the former was more limited. Numerous studies have demonstrated that the quality of life of the nasal sinuses is minimally or not significantly altered following neuroendoscopic transnasal transsphenoidal approach pituitary resection. Although the submucosal approach of the nasal septum elicits more pronounced early nasal sinus symptoms, questionnaire results are generally comparable (17–19). Zhu et al. conducted a systematic analysis of the clinical outcomes of 57 patients who underwent the septum mucosal flap approach. The incidence of olfactory dysfunction was 28% two weeks postoperatively; however, most symptoms were either substantially alleviated or resolved within three months (5).

In this research, we analyzed two surgical methods for nasal mucosa incision to dynamically assess their impact on postoperative olfactory function. There was no significant difference between the two groups in olfactory recognition threshold at various time intervals postoperatively. However, when compared to the preoperative values, the olfactory recognition thresholds at one, three, and six months after surgery were statistically significant in both groups based on the five-flavor olfactory test. Nevertheless, the recovery of the sense of smell exhibited a gradual and continuous improvement within the first six months after surgery. The two groups were compared for five odors at varying time intervals after surgery. At one and three months following surgery, the submucosal group demonstrated a greater sensitivity to offensive odor.

This study confirmed that patients who underwent neuroendoscopic transnasal transsphenoidal resection of pituitary adenomas experienced varying degrees of olfactory dysfunction following the procedure. The nasal septum mucosal incision in the submucosal approach substantially improved early odor recognition. Diverse mucosal incision methods demonstrated comparable effects on the long-term olfactory function of patients following the procedure. Limitations of this study should be acknowledged. First, this retrospective single-center study lacked randomized assignment of surgical approaches, and surgeons chose mucosal incision methods based on personal experience rather than standardized tumor stratification criteria. However, balanced baseline characteristics between groups greatly reduced confounding bias caused by different tumor burdens. Second, only subjective olfactory examinations were applied without objective electrophysiological olfactory detection, and the follow-up duration was limited to 6 months. Long-term multicenter prospective studies are required to validate our conclusions.

## Data Availability

The original contributions presented in the study are included in the article/supplementary material, further inquiries can be directed to the corresponding author.
